# Graphene based electrochemical immunosensor for the ultra-sensitive label free detection of Alzheimer's beta amyloid peptides Aβ(1–42)†

**DOI:** 10.1039/d0na00801j

**Published:** 2021-02-20

**Authors:** Hina. Y. Abbasi, Zari Tehrani, Anitha Devadoss, Muhammad Munem Ali, Soraya Moradi-Bachiller, Diego Albani, Owen. J. Guy

**Affiliations:** Centre for NanoHealth, College of Engineering, Swansea University Swansea SA2 8PP UK h.y.abbasi@swansea.ac.uk o.j.guy@swansea.ac.uk +44 (0) 1792 606475 +44 (0) 1792 513181; Department of Chemistry, College of Science, Swansea University Swansea SA2 8PP UK; Department of Neuroscience, Istituto di Ricerche Farmacologiche Mario Negri IRCCS Via La Masa 19 20156 Milan Italy

## Abstract

An immunosensor capable of high sensitivity detection of beta-amyloid peptides, shown to be a reliable biomarker for Alzheimer's disease, has been developed using screen printed graphene electrodes (SPGEs) modified with ultra-thin layers of polymerised 1,5-diaminonaphthalene (pDAN). Electropolymerization of 1,5-diaminonaphthalene (DAN) was performed to coat the graphene screen printed electrodes in a continuous polymer layer with controlled thickness. The surface characteristics of pristine graphene and polymer modified graphene electrodes were examined using Raman and X-ray photoelectron spectroscopy. The effects of polymer thickness on the electron transfer rates were investigated. An immunosensor for selective detection of beta amyloid peptides Aβ(1–42) was developed *via* biofunctionalization of the pDAN modified SPGE with the anti-beta amyloid antibody used as the peptide bioreceptor. The immunosensor has been used for specific detection of Aβ(1–42) with a linear range of 1 pg mL^−1^ to 1000 pg mL^−1^ and showed 1.4 pg mL^−1^ and 4.25 pg mL^−1^ detection and quantification limit, respectively. The biosensor was further validated for the analysis of spiked human plasma. The immunosensor enables rapid, accurate, precise, reproducible and highly sensitive detection of Aβ(1–42) using a low-cost SPGE platform, which opens the possibilities for diagnostic *ex vivo* applications and research-based real time studies.

## Abstract 2

Development of ultra-thin polymer layer modified screen printed graphene electrodes with high conductivity for higher-sensitivity detection of Aβ(1–42) peptides with the lowest LOD of 1.4 pg mL^−1^ is presented.

## Graphical abstract

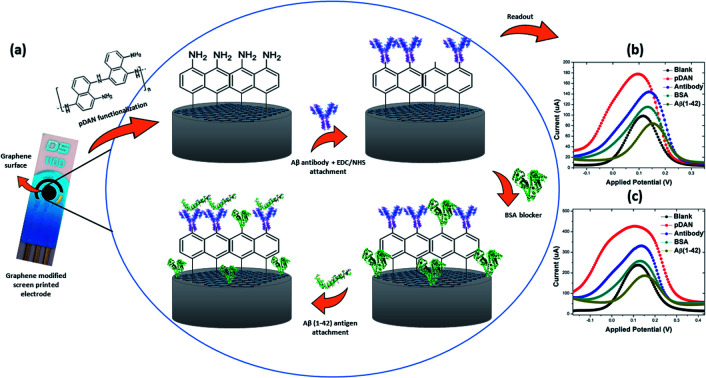

## 1. Introduction

Alzheimer's Disease (AD) is the most common cause of dementia that influences millions of people across the world and becomes more prevalent with aging. AD is a neurodegenerative disease triggered by extracellular accumulation of amyloid β peptide (Aβ), intracellular appearance of neurofibrillary tangles and neuronal loss.^1,2^ This degeneration leads to changes in behaviour, personality and functional capacity, which deter the daily life of the patient. In 2020, an estimated 35 million people suffered from AD globally and it is predicted to affect 115 million individuals by 2050.^3^ The existing investigation methods of AD are complicated and are usually made when the disease is already in an advanced stage. In addition, there are no treatments available to avoid this condition^4^ and current therapies only slow the advancement of the disease.^4^ Thus, there is an intense need for the development of easy analytical tools for the rapid detection of AD biomarkers for early-stage point-of-care diagnosis.^5–7^ Aβ(1–42) peptide is the key element of the senile plaques present in AD.^8^ Other pathological characteristics of AD consist of intraneuronal inclusions of hyperphosphorylated tau protein in neurofibrillary tangles, together with downstream processes such as inflammation and oxidative stress. Additionally, a certain isoform of apolipoprotein, ApoE4, is the foremost genetic risk factor for AD, because it also leads to excess of amyloid formation in the brain.^9^ All these components cause the loss of synaptic integrity, progressive neurodegeneration and effective neural network connectivity.^3,6,10^

Aβ(1–42) is usually expressed in cerebrospinal fluid (CSF) and plasma and it is found that the CSF levels of Aβ(1–42) are lower in AD patients than in normal controls, which indicates amyloid pathology. Gagni *et al.* reported the detection of low CSF Aβ(1–42) levels at preclinical disease stages that predicted future cognitive decline and neurodegeneration.^11^ An Aβ(1–42) concentration of <500 pg mL^−1^ (0.1 nM) indicates that Aβ(1–42) is accumulating in the brain and not circulating in the CSF.^11^ However, more recently Emadi *et al.* reported that Aβ(1–42) concentration levels in peripheral blood serum samples at the preclinical disease stage is 3.41 ± 2.17 pg mL^−1^ in aged people and 3.34 ± 1.95 pg mL^−1^ in young people.^12^ They stated that in female AD patients with increasing age, more amounts of Aβ(1–42) remain in brain and thus appears less in the patient's serum, whereas in male AD patients the amount of Aβ(1–42) that remains in the brain is smaller than that of Aβ(1–40). It was concluded that female AD patients would show more adverse cognitive decline than male patients with increasing age because the toxicity of Aβ(1–42) has been shown to be far more than that of Aβ(1–40).^12^ It is therefore suggested that detection of Aβ(1–42) in physiological fluids such as CSF, serum and plasma at lower levels can be used for both screening of AD at an early stage and for monitoring disease progression.

Several methods have been introduced to detect amyloid beta using different sensing platforms.^13–19^ An enzyme linked immunosorbent assay (ELISA), which is evaluated by western or dot blot analysis, is currently used for its clinical detection. Although these tests are reliable, they are labour intensive, time consuming and require complicated instruments to perform measurements. Also, the sensitivity of these tests is not adequate to detect the ultra-low levels of the disease biomarkers at the early stages of disease onset.

In 2013 Jeseung *et al.* reported a CNT film-based biosensor with a metal semiconductor field effect transistor structure (CNT-MESFET) for the real time detection of amyloid-β (Aβ) in human serum. Their sensor showed an LOD of 1 pg mL^−1^ within the linear range of 10^−12^ to 10^−9^ g mL^−1^. Nevertheless, the fabrication of this sensor is complex, costly and labour-intensive.^15^

Electrochemical biosensors offer a rapid, cost-effective, easy and sensitive testing technique.^10,14,16,20,21^ Furthermore, label-free electrochemical immunosensors reduce sample complexity because of the exclusion of potentially confounding molecular labels.^22,23^ They provide a promising approach for both sensitive and selective analysis due to their high compatibility and repeatability, rapidness, simple instrumentation, low power requirements and easy signal quantification.^24–26^ Label-free sensors on the other hand can also be integrated into lab-on-a-chip platforms and have the benefit of using small volumes for rapid and inexpensive measurements as opposed to the label-based technologies which are often more costly and time-consuming.^27^ Pedro and co-workers used a modified gold electrode surface for the sensitive detection of Aβ(1–42). This sensor showed good accuracy and reproducibility with a limit of detection of 5.2 pg mL^−1^. However, the selected antibody was also able to recognize Aβ(1–40), limiting the specificity of the technique.^16^ Furthermore, Troung *et al* presented a label-free impedimetric immunosensor using carbon disposable electrochemical printed chips modified with AuNPs, for the detection of amyloid beta. They bound protein G to the antibody for its controlled immobilization which in turn lowered the LOD to 0.57 nM.^21^

It has been reported that coating polymeric films on the electrode surface may well improve the behaviour and performance of electrochemically modified electrodes.^28,29^ Furthermore, such modifications may increase the reaction rate, enhance the electrocatalytic properties of the substrates, and the reproducibility and stability of the electrodes.^30^ Poly(1,5diaminonaphthalene) (pDAN) is a conducting polymer that is obtained from the polymerization of aromatic monomers comprising of two amino groups.^31^ Thus, there are free amine groups present in the polymer structure that aid in binding the biomolecules to the electrode surface. The unique properties of pDAN make it an outstanding material for electrochemical electrode modification. Several studies have used pDAN in electrochemical sensors for detection of, for example, H_2_O_2_, cholesterol, H_2_O, dopamine and lactose.^32–36^

In this work we have used a graphene based electrode, due to its high conductivity, large surface to volume ratio, low cost and low environmental impact, in the fabrication of sensors and biosensor-based devices.^37,38^ Graphene biosensors offer the advantages of high sensitivity, lower detection limits and high throughput detection when compared to other methods such as ELISA, Polymerase Chain Reaction (PCR) and fluorescence assays. Myung-Sic *et al.* used oxygen-plasma-treated rGO surfaces as reactive interfaces for the electrical detection of Aβ peptides, which in turn improved the antibody immobilization on electrodes and yielded improved sensing performance due to the enhancement in surface functionality. This was confirmed by measuring the changes in the electrode's electrical characteristics, with a 3.33-fold steeper slope for electrical responses *versus* the analyte concentration curve of the oxygen plasma treated sample compared to the untreated one.^39^ More recently a graphene/rGO dual layer SPE was reported by Sethi and co-workers for the detection of Aβ(1–42). The proposed sensor showed a detection limit of 2.398 pM with high selectivity.^40^

Here, we report the development of a label-free, electrochemical immunosensor, using graphene modified screen printed electrodes (SPGEs) for high sensitivity detection of beta amyloid peptides, isoform 42. The graphene surface was modified with amine functional groups using a 1,5-diaminonaphthalene (DAN) electropolymerization process with optimised amine surface coverage,^41^ whilst maintaining minimal thickness for better charge transfer from the electrolyte solution to the electrode surface. Polymer DAN (pDAN) modification provided controlled amount of amines without even compromising the actual graphene properties. Subsequent attachment of the anti-beta amyloid antibody on to the sensor was performed and the influence of antibody concentrations with respect to Aβ(1–42) peptide sensing efficiency has been analysed in detail. BSA was used as the blocking agent. The implications of this work towards developing a commercially viable, robust and sensitive immunosensor for Alzheimer's disease by using biomarker Aβ(1–42) are presented.

## 2. Materials and methods

### 2.1. Materials

Graphene modified screen printed electrodes (SPGEs) were purchased from Metrohm Ltd; 1,5-diaminonaphthalene (DAN) (97%), 1-ethyl-3-(3-(dimethylamino)-propyl) carbodiimide hydrochloride (EDC) (>98%), *N*-hydroxysuccinimide (NHS) (98%), ferrocene carboxylic acid (FeCOOH) (97%), potassium hexacyanoferrate(iii) (>99%), potassium hexacyanoferrate(II) trihydrate (98.5–102%) and phosphate buffered saline (PBS) tablets were supplied by Sigma-Aldrich and used as received. Anti-beta amyloid antibody and beta-amyloid peptide (1–42) human were obtained from Abcam Ltd.

### 2.2. Electropolymerization

Graphene electrode surfaces were functionalized with ultra-thin polymer layers of 1,5-diaminonaphthalene (DAN) *via* an electropolymerization technique mentioned in detail in our previous work.^41^ Typically, the polymer films were deposited from 10 mM DAN in 0.25 M H_2_SO_4_ using standard graphene modified screen printed electrodes (SPGEs), where graphene was used as the working electrode, carbon as the counter electrode and Ag/AgCl as the reference electrode. Electropolymerization of the monomer DAN results in the deposition of ultra-thin polymer DAN (pDAN) layers on the graphene electrode surface, with the resulting pDAN layer containing NH_2_ functional groups which can subsequently be used for the attachment of “bioreceptor” antibodies to the graphene surface. Following pDAN deposition, the electrodes were rinsed with deionized (DI) water and dried under nitrogen.

### 2.3. Bio-functionalization

Bio-functionalization protocols were optimised for improved sensor performance. Typically, anti-beta amyloid antibody was ‘activated’ in a solution containing 5 mM EDC/NHS for 40 minutes. Once activated, the antibody solution was added to the sensor surface and incubated for 30 minutes at 4 °C. The antibody incubation time was optimised to achieve the highest sensitivity for the proposed biosensor (Fig. S1†). The antibody functionalised SPGE was then rinsed thoroughly with deionised water, to wash away any non-specifically bound probe on the surface. Blocking of unbound NH_2_ surfaces was performed by drop-casting 1% BSA onto the electrode surface and incubating for 30 minutes at 4 °C, subsequently removing any unbound BSA by rinsing with DI water and drying with N_2_. In this work 0.1 mM concentration of PBS was used in all experiments in order to increase the Debye length to approximately 7.3 nm and hence to achieve better signal amplification.^42^

### 2.4. Amyloid beta (1–42) pre-treatment method

Aβ(1–42) was received in the lyophilized form. It was first dissolved in 10 mM sodium hydroxide, followed by gentle vortexing for less than 1 minute to make a homogeneous solution. It is reported that under these highly alkaline conditions the peptide is fully dissolved and exists only as monomers.^43^ The stock solution was then aliquoted and stored at −20 °C until further use.

### 2.5. Human blood collection and plasma preparation

Human plasma samples were received from IRCCS – Istituto di Ricerche Farmacologiche “Mario Negri” *Via* La Masa 19, 20 156 Milan – Italy. Fasting blood samples (3 mL) from AD patients were collected by the venipuncture method. The aliquot was centrifuged at 2000 rpm for 15 minutes at 4 °C to separate the plasma fraction. After that the plasma was transferred to a fresh tube, aliquoted and immediately frozen at −80 °C till further use. Informed consent was obtained from all human subjects. Besides, all of the investigation protocols in this study have been approved by relevant local ethics committee for clinical research (Milan, Italy).

### 2.6. Characterization

The electrode layers were characterized for their thickness using Raman spectroscopy and X-ray photoelectron spectroscopy to check the quality of the electrode.

X-ray photoelectron spectroscopic (XPS) measurements were performed using a Kratos Axis Supra, with an Al Kα monochromatic X-ray source, running at an emission current of 15 mA. Raman mapping measurements were performed using a Renishaw system, with a 532 nm excitation laser and 2 mW power, before and after pDAN modification on the electrode surface.

Electrochemical analysis was done using an advanced potentiostat (PGSTAT-302N, Metrohm AutoLab, Runcorn, UK). Square wave voltammetry (SWV) and differential pulse voltammetry (DPV) were used as characterization techniques with the scanning voltage in the range of −0.2 V to 0.5 V for evaluating the electrochemical performance of blank and modified electrodes. 5 mM K_3_[Fe(CN)_6_]/K_4_[Fe(CN)_6_] in 0.1 mM PBS solution (pH = 7.4) was used as an electrolyte.

## 3. Results and discussion

### 3.1. Grafting pDAN layers on SPGEs

To enable biofunctionalization, SPGE surfaces were modified with poly-1,5-diamino naphthalene (pDAN) layers using electropolymerization as described in Section 2.2. Fig. S2† shows the voltammetric response of SPGEs during the electropolymerization process. The dominant peak at 0.55 V (*vs.* Ag/AgCl) corresponds to the oxidation of the monomer DAN species, which reduces subsequently with increase in the number of scan cycles, indicating that DAN monomers are being oxidised and converted into polymers. Also, a new peak at 0.3 V originating during the second cycle is attributed to polymer layer formation and this peak increases with increase in the scan cycles. Polymer layer thickness was controlled *via* controlling the number of scan cycles. In order to achieve a greater number of surface amine groups for biofunctionalization, SPGE electrodes were modified with pDAN layers of different thicknesses.^41^

### 3.2. Mechanism of action of pDAN/graphene interfaces

#### 3.2.1. Electrochemical analysis

The electroactivity of the deposited pDAN films was studied using cyclic voltammetry. Fig. S3† shows cyclic voltammetry curves of pDAN layers in 5 mM Fe^2+^/Fe^3+^ redox couple at the scan rates of 10–100 mV s^−1^ (discussed in detail in Section 3). To further analyze these systems, the charge transfer diffusion coefficient, *D*_CT_, was calculated using the Randles–Sevcik equation (eqn (1)):

1 *I*_p_ = (2.69 × 10^5^)*n*^3/2^*ACD*^1/2^*υ*^1/2^

where *I*_p_ represents the peak current, *A* is the area of the electrode (0.1256 cm^2^), *n* is the number of electrons transferred (*n* = 1), *C* is the concentration of the redox species (5 mM), *D* is the charge transfer diffusion coefficient (*D*_CT_) and *υ* is the scan rate. The blank graphene electrode displayed a typical *D*_CT_ of 13.2 × 10^6^ cm^2^ s^−1^ due to its high electronic mobility. The charge transfer diffusion coefficient was highest (6.80 × 10^6^ cm^2^ s^−1^) during the first cycle of pDAN deposition, which was as expected, as the single layer of monomer DAN (Fig. S5†) allows the most facile charge transfer to the graphene surface. With increase in pDAN layer thickness to 2 cycles *D*_CT_ decreases to 4.88 × 10^6^ cm^2^ s^−1^ and again increases at its maximum (5.87 × 10^6^ cm^2^ s^−1^) at 3 cycles indicating that a uniform pDAN monolayer is achieved, followed by a decrease again to 3.29 × 10^6^ cm^2^ s^−1^ and 1.03 × 10^6^ cm^2^ s^−1^ at 5 and 10 cycles, respectively. This shows that the electron transfer rates are affected by the thickness of the pDAN layer and thus, the pDAN layer should be optimized in terms of maximizing the number of surface NH_2_ groups, whilst maintaining the highest possible electron transfer rates.

#### 3.2.2. Quantification of amine surface groups

**Fig. 1:**
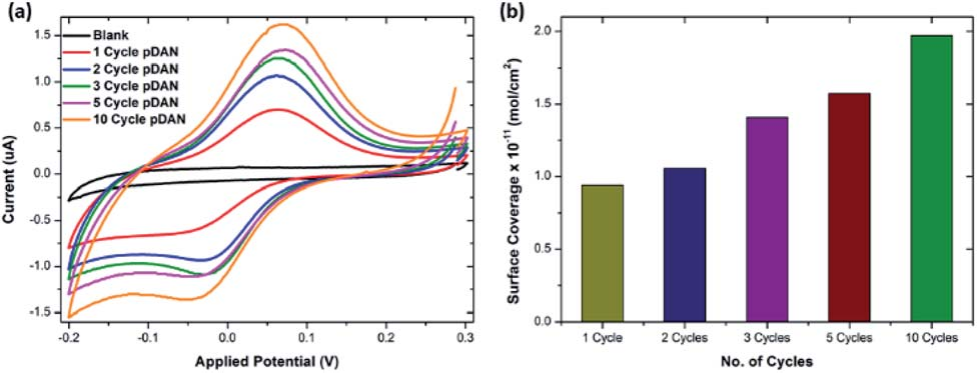
(a) Cyclic voltammogram of ferrocene carboxylic acid functionalized SPGE and pDAN modified SPGEs at different scan cycles. PBS was used as the electrolyte and the CVs were recorded at a 5 mV s^−1^ scan rate. (b) Bar chart showing amine surface coverage *vs.* the number of pDAN scan cycles.

The efficiency of chemical functionalization was analyzed by estimating the number of surface amine groups upon pDAN modification *via* functionalizing the DAN-modified electrodes with ferrocene carboxylic acid (FCA), using a standard EDC/NHS coupling reaction. Fig. 1a shows the cyclic voltammogram of FCA functionalized graphene and pDAN modified SPGEs. The electrodes were scanned between −0.2 V and 0.3 V at 5 mV s^−1^ scan rate. No redox peak is found for blank SPGE upon FCA functionalization due to the lack of amine functional groups present on the surface, whereas at 0.07 V and −0.04 V strong redox peaks are observed for FCA functionalized pDAN modified SPGEs, which correlate with the oxidation and reduction of the ferrocene moiety, respectively. The ferrocene surface coverage was estimated using eqn (2):

2 *Γ* = *Q*/*nFA*

where *Q* is the charge obtained by integrating the anodic peak at a low scan rate (coulomb), *n* is the number of electrons transferred, *F* is the Faraday constant (96 485 s A mol^−1^) and *A* is the geometrical area of the electrode (0.1256 cm^2^). Fig. 1b shows the surface coverage dependence on the number of scan cycles. As anticipated, it is found that the amine surface coverage increases with increase in pDAN layer thickness (scan cycles), which is also attested by the increase in the capacitive current. Although the higher pDAN layer thickness produces large amine density on the surface, it is found that the charge transfer characteristics decrease after reaching a certain thickness (Section 3.2.1). Thus, it is critical to find a fair balance between increased amine surface coverage as well as charge transfer characteristics. For our experiments – 3 cycles of pDAN layers (3-pDAN) were chosen to be the optimal layer thickness for further electrochemical sensor development.

#### 3.2.3. Structural and chemical analysis

**Fig. 2:**
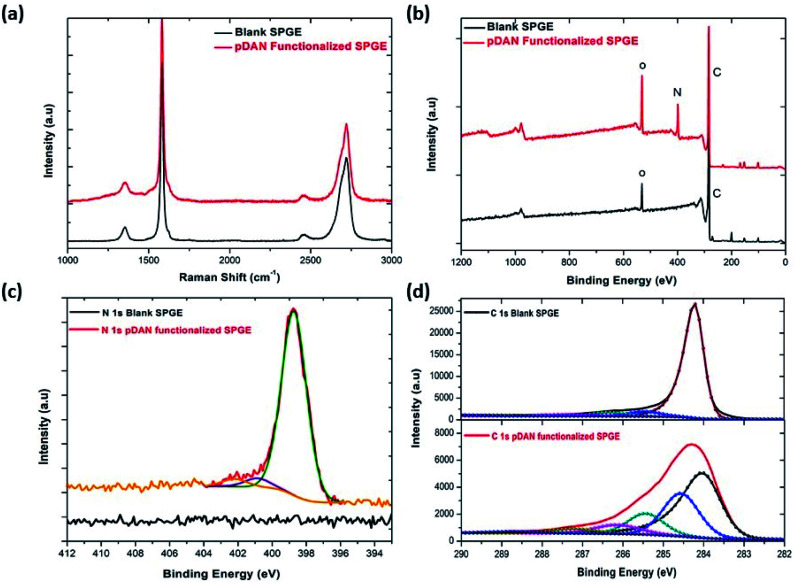
Comparison of blank and pDAN functionalized graphene SPE at 3 scan cycles (a) Raman spectrum; (b) XPS spectrum showing wide scan, pDAN functionalized SPGE showing (c) N 1s spectrum (d) C 1s spectrum.

Raman spectroscopic analyses of pristine SPGEs and pDAN-modified SPGEs are shown in Fig. 2a. The Raman spectrum of pristine SPGE is similar to that of pristine graphene, showing the D, G and 2D band positions at 1350 cm^−1^, 1580 cm^−1^ and 2700 cm^−1^ respectively.^44^ The corresponding intensity ratio obtained from G and 2D bands of the pristine SPGEs was 0.46633, which confirms the presence of the few layer graphene structure on the screen-printed electrode. After pDAN modification, the intensity ratio of G and 2D bands of the pDAN modified SPGE was found to be 0.42407. The intensity ratios of the D and G bands of the pristine and pDAN modified SPGEs were calculated to be 0.46633 and 0.42407, respectively. Taking device variation into consideration, no significant variations were found in the intensity and position of the D, G and 2D bands upon pDAN modification, alluding that very little structural deformation is induced to the graphene upon electropolymerization.

X-ray photoelectron spectroscopy is a tool used to investigate the chemical environment of graphene modified with foreign dopants.^41,45^ To study the influence of electropolymerization on SPGEs, XPS measurements were carried out on blank and pDAN modified SPGE surfaces. Fig. 2b–d show XPS graphs of the wide spectrum (b), N 1s spectrum (c) and C 1s spectrum (d) of pristine graphene and pDAN modified SPGEs. The wide scan acquisition of pristine and pDAN modified SPGE shows a typical C 1s peak at ∼284 eV. Unlike pristine graphene, the pDAN modified SPGE shows a clear peak at ∼400 eV in the wide spectra (Fig. 2b), confirming that nitrogen-based moieties were introduced after electropolymerization. The atomic concentrations of C, O and N present were calculated from the XPS spectra and are listed in Table S2.† It is found that the atomic percentage of N increases drastically upon electropolymerization and additional components appear in the C spectra (Fig. 2d), which can be ascribed to the carbon and nitrogen species present in DAN. To further analyse the nature of C, O, and N species, the N 1s (Fig. 2c) and C 1s (Fig. 2d) peaks were fitted with Gaussian/Lorentzian peaks to analyse the distribution of carbon and nitrogen bonding. The C 1s peak of pDAN modified graphene was deconvoluted into six carbon peaks, which are ascribed to C_aromatic_ (∼284.04 eV), C_aliphatic_ (∼284.56 eV), C–N (∼285.44 eV), C–O (∼286.14), C=O/C=N (∼287.20 eV) and O–C=O/N–C=O (∼288.76 eV). The N 1s peak of pDAN modified graphene is observed at 400.16 eV, indicating that nitrogen compounds are present upon successful electropolymerization. Furthermore, the three fitted peaks at ∼400.12 eV, 402.26 eV and ∼403.59 eV correspond to C–N, C–NH and N–H groups respectively, confirming that the pDAN electropolymerization process generates –NH_2_ groups on the graphene surface.^46^

### 3.3. Mechanism of antibody/pDAN/graphene interfaces

#### 3.3.1. Electrochemical analysis

**Fig. 3:**
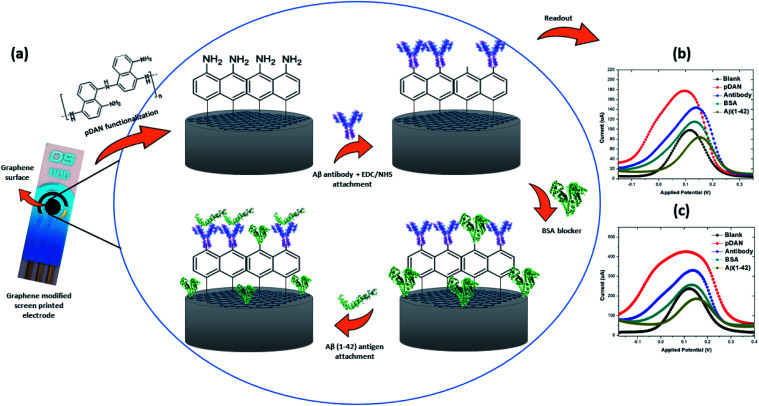
(a) Schematic representation of the electrochemical immunosensor showing changes on the surface after each functionalization stage; (b) differential pulse voltammetry (DPV) response; (c) square wave voltammetry (SWV) response.

A carboxyl terminated anti-beta amyloid antibody specific to Aβ(1–42) peptide was immobilized onto the pDAN modified SPGE surface *via* carbodiimide linkage using an EDC/NHS protocol.^47^Fig. 3 (a) shows the schematic representation of the sensor while (b) shows the differential pulse voltammetric (DPV) response and (c) the square wave voltammetric (SWV) response of SPGE at each stage of functionalization. An increase in the peak current is observed upon pDAN functionalization, which could be attributed to the increased charge transfer at the electrode/electrolyte interface due to the high conductivity of the amine (NH_2_) layer.^48^ Slight peak broadening is also observed which may be due to the non-uniform coating of pDAN layers. The subsequent antibody functionalization reduces the available sites for charge transfer *via* blocking, and thus a drastic reduction in the peak current is observed. This shows that the antibody functionalization impedes the charge transfer process at the electrode/electrolyte interface. Similarly, blocking of unbound NH_2_ sites at the electrode surface using BSA further reduces the peak current, suggesting the successful biofunctionalization of pDAN modified SPGEs with antibody and BSA.

#### 3.3.2. Optimising the antibody concentrations

**Fig. 4:**
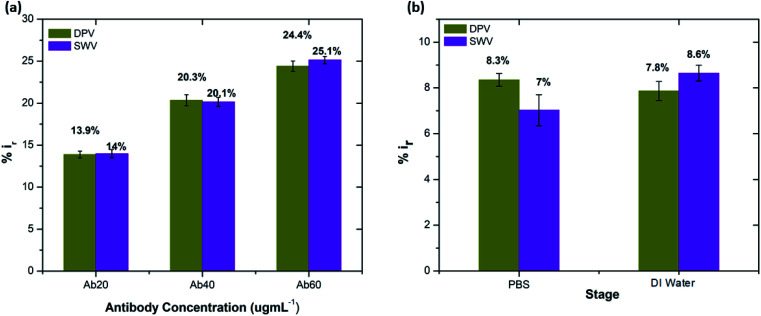
(a) Peak current reduction in DPV and SWV measurements (%*i*_r_) at each concentration of Aβ antibody. (b) Percentage reduction in peak current on exposure to DI water and PBS (0.1 mM) (*n* = 3).

The concentration of the biomolecules immobilised onto the electrode surface greatly influences the immunosensor performance. Thus, optimising the antibody concentration is critical to ensure the superior performance of the electrochemical immunosensor. In this work, we have investigated the influence of the immobilised antibody concentrations on sensing. A controlled immobilization of anti-beta amyloid antibody onto pDAN modified SPGEs was achieved by varying the antibody concentrations (20, 40 and 60 μg mL^−1^). Electrochemical analysis of the antibody attached SPGEs was performed using DPV and SWV, respectively. It is observed that the peak current produced *via* the electron transfer upon the [Fe(CN)6]^3−/4−^ redox process is greatly influenced by the amount of antibody immobilised onto the electrode surface. Fig. 4a shows the dependence of the percentage of peak current reduction on the concentration of the antibodies. It is evident that 60 μg mL^−1^ Aβ antibodies reduce the current by ∼25% whereas 40 μg mL^−1^ antibodies reduce the current by ∼20% and a much lower level of peak current reduction by ∼14% is observed with 20 μg mL^−1^ anti-beta amyloid antibodies. This could be clearly attributed to the increased antibody surface coverage at the electrode which could increase the steric barrier for the [Fe(CN)6]^3−/4−^ redox system in accessing the electrode surface – resulting in decreased peak current generation.

#### 3.3.3. Optimising the sensor for false-positives

**Fig. 5:**
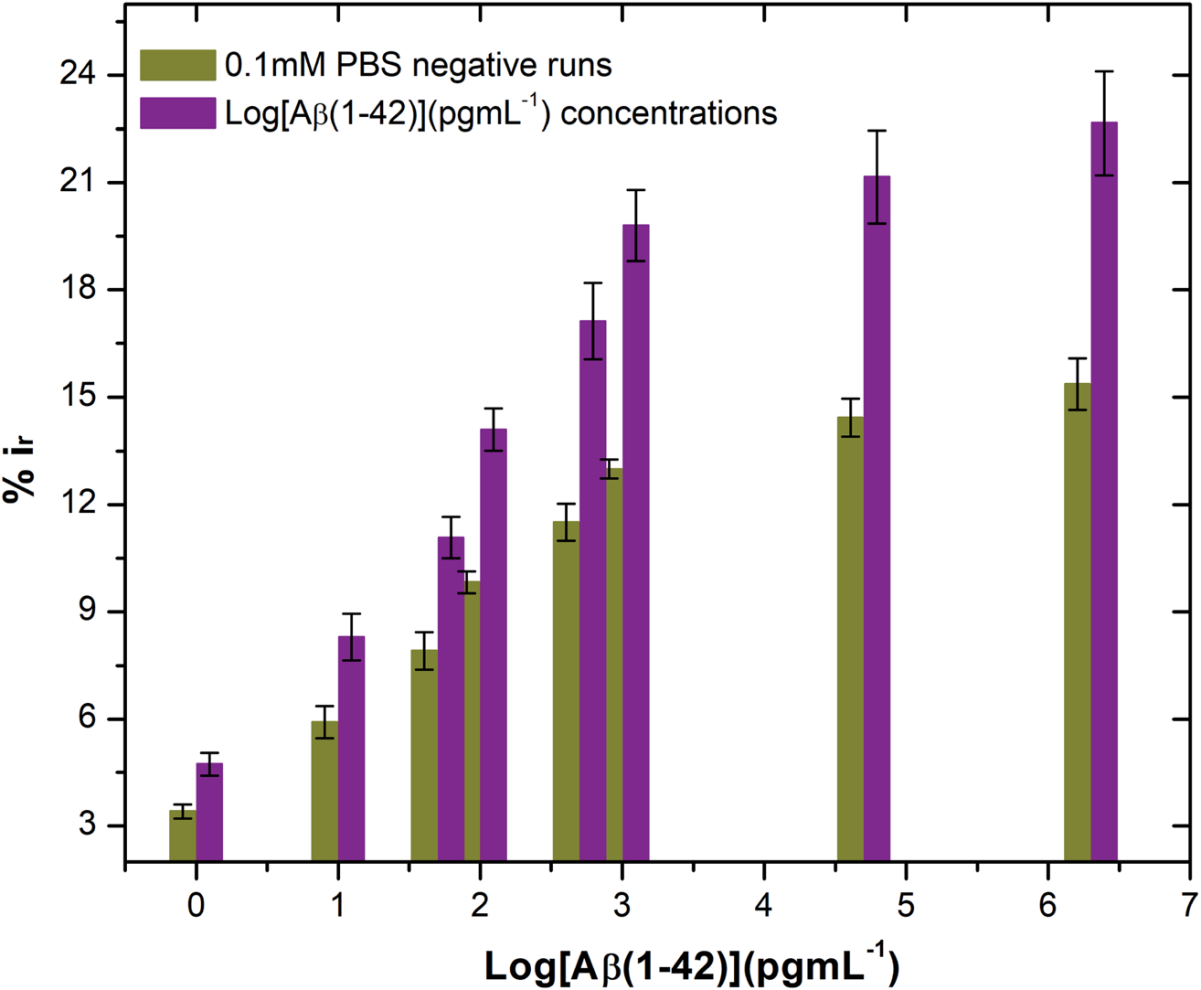
Bar chart illustrating the peak current reduction (%*i*_r_) with exposure to 0.1 mM PBS only (negative runs with respect to each peptide concentration) and log of different concentrations of beta amyloid peptide Aβ(1–42) (*n* = 3).

Analysis of the effect of sensor response to false positives, *i.e.*, to PBS and DI water was performed by considering the effect of 0.1 mM PBS and DI water exposure on the sensors (antibody functionalized SPGEs). Exposure to PBS and DI water was repeated multiple times. Fig. 4b shows the percentage change in the peak current upon exposing the sensors to 0.1 mM PBS and DI water. A considerable change (∼7–8%) in the peak current is observed upon exposure to 0.1 mM PBS. A similar effect is observed for DI water exposure, which could be ascribed to the high sensitivity of the graphene-based electrode surface. The relative current change corresponding to 0.1 mM PBS and beta amyloid peptides Aβ(1–42) binding to the antibody/BSA-bound surface was estimated using eqn (3):^49^3
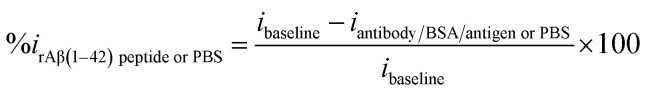
where *i*_baseline_ and *i*_antibody/BSA/antigen or PBS_ are the mean DPV currents obtained at unmodified (blank) and antibody/BSA/Aβ(1–42) antigen or PBS modified electrodes, respectively. It is found that antibody functionalised electrodes show significant change in the peak current with exposure to the relative peptide compared to changes observed on exposure to 0.1 mM PBS only (Fig. 5). The repeated exposure of antibody functionalised SPGEs to 0.1 mM PBS only showed reproducible changes in current. However, the current change was minimal (1–1.5%) when compared to the change measured upon peptide binding. Thus, to moderate the effect of PBS, every sensor was exposed to 0.1 mM PBS first before performing the actual peptide sensing measurement in order to saturate the electrode surface with PBS.

### 3.4. Evaluating the electrochemical immunosensor performance

#### 3.4.1. Sensitivity analysis

**Fig. 6:**
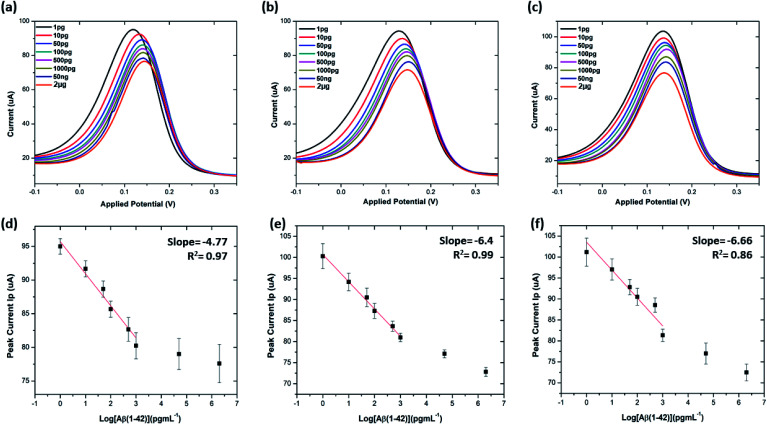
Differential pulse voltammetry (DPV) results at 20 μg mL^−1^, 40 μg mL^−1^ and 60 μg mL^−1^ concentration of Aβ antibodies: (a–c) DPV plots of 20 μg mL^−1^, 40 μg mL^−1^ and 60 μg mL^−1^ antibodies at each stage of sensing; (d–f) corresponding calibration curves of Ip as against log Aβ(1–42) concentrations. All data points are mean values of three independent electrodes. The error bars (calculated as the standard deviation) provide a measure of repeatability of the system (*n* = 3).

The electrochemical immunosensor performance was evaluated by exposing the antibody functionalised electrodes to different concentrations of beta amyloid peptides Aβ(1–42) ranging from 1 pg mL^−1^ to 2 μg mL^−1^ at 4 °C, each for incubation times of 20 minutes. This was expected to result in the successful binding of the beta amyloid peptides Aβ(1–42) to the anti-beta amyloid antibodies, which would in turn affect the [Fe(CN)6]^3−/4−^ electron transfer. It is found that the peak current decreased significantly with an increase in the peptide concentration from 1 pg mL^−1^ to 2 μg mL^−1^. This is because increasing the concentration leads to high accumulation of proteins on the electrode that in turn blocks the [Fe(CN)6]^3−/4−^ redox system from accessing the electrode surface. Saturation of the electrode surface is observed at >1000 pg mL^−1^ peptide concentrations with 20 μg mL^−1^ antibody electrode. Fig. 6 shows the sensor performance and calibration curve of peak current variation *vs.* log of concentration of the beta amyloid peptides for SPGEs modified with antibody Aβ(1–42) concentrations of 20, 40, and 60 μg mL^−1^, respectively. The calibration curve shows a linear relationship between the peak current and log[peptide concentration]. Our electrochemical sensor showed a wide linear response between 1 pg mL^−1^ and 1000 pg mL^−1^ (Fig. 6d–f).

The limit of detection (LOD) and limit of quantitation (LOQ) for the precise detection of amyloid β peptide (1–42) were calculated within the linear range of 1 pg mL^−1^ to 1000 pg mL^−1^, utilising the standard deviation of the intercepts and the average of slopes of the straight lines from the analytical curves, using the following equations:^16,40,52,53^

4 LOD = 3.3(SD of the lowest concentration/SLOPE)

5 LOQ = 10(SD of the lowest concentration/SLOPE)

where SD is the standard deviation of the predicted peak current values, and SLOPE is the slope of the calibration line.

Table S3† shows the list of LODs and LOQs obtained for the electrodes functionalised with different concentrations of antibody. Sensors functionalised with 20 μg mL^−1^ antibody show the lowest LOD, 1.4 pg mL^−1^ and 4.25 pg mL^−1^ LOQ. It is observed that the LOD increased with increase in the concentration of the surface bound antibodies, indicating the significance of achieving an optimal antibody loading onto the sensor surface. Our sensor shows higher sensitivity over the other existing label-free biosensors (Table 1).

**Table 1:** An overview of recently reported label-free biosensors for Aβ(1–42) detection^a^

Electrode materials	Receptor system	Detection techniques	Limit of detection (pg mL^−1^)	Ref.
Au	Anti-Aβ(1–42)/AuNPs/MPA/Au electrode	EIS, SWV	5.2	16
SiO_2_	Anti-Aβ(1–16)/glutaraldehyde/PVP-CHO/3-aminopropyl triethoxysilane	Electrical impedance	—	50
Graphene/rGO SPE	Anti Aβ(1–42) antibody (H31L21)/Pyr-NHS/graphene-rGO SPE	DPV	10.8*	40
Si/SiO_2_	(Range of antibodies**)/poly(DMA-*co*-NAS-*co*-MAPS)/silicon microarrays	Fluorescence	73	11
Carbon ink electrode of DEP chip	Monoclonal Aβ antibody/protein G/MHDA SAM/AuNPs/carbon DEP chip	EIS	2573.02*	21
ICE-Au	Anti-Aβ antibody (MOAB-2)/EDC-NHS/MHA SAM/ICE	EIS	70, 100	51
Graphene SPE	Anti Aβ(1–42) antibody (mOC64)/pDAN/graphene SPE	DPV	1.4	This work

a *Values were converted from pM and nM to pg mL^−1^. **SC-D17, NT-11H3, NT-8G7, Cov-4G8 and Cov-12F4.

#### 3.4.2. Specificity and stability analysis

**Fig. 7:**
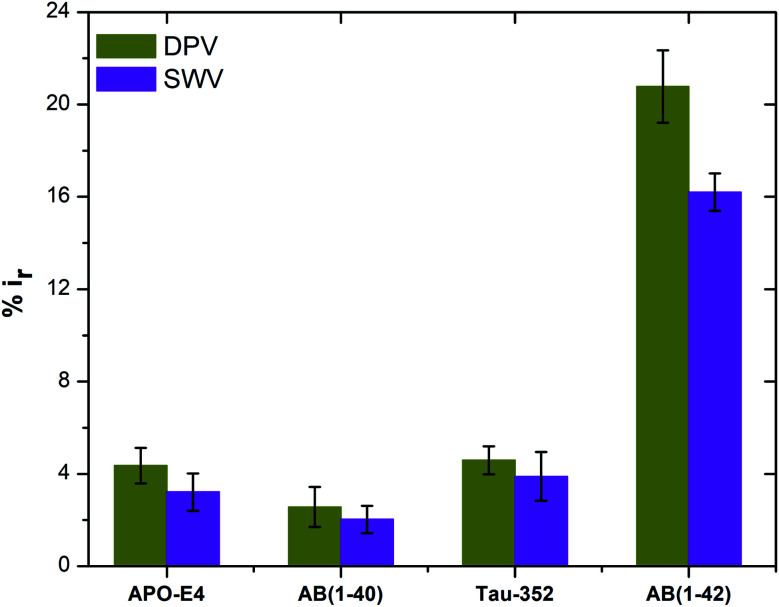
Peak current reduction %*i*_r_ obtained from DPV and SWV plots for non-specific testing using APO-E4, Aβ(1–40), Tau-352 and Aβ(1–42) (*n* = 3).

To test the selectivity and specificity of our sensors for Aβ(1–42) detection, the antibody immobilised sensors were incubated with 1000 pg mL^−1^ concentrations of APO-E4, Aβ(1–40), Tau-352 and Aβ(1–42) proteins, respectively. Fig. 7 shows the percentage change in the peak current (DPV measurements) upon binding with APO-E4, Aβ(1–40), Tau-352 and Aβ(1–42). A very small change (2–4.5%) in peak current is observed for APO-E4, Aβ(1–40) and Tau-352 proteins compared to Aβ(1–42) protein (16–21% change). The small changes on exposure to non-specific proteins may be because of graphene's sensitivity towards PBS (as discussed in Section 3.3.3.) and some non-specific adsorption of the peptides onto the sensors. In contrast, a very strong change is observed after addition of Aβ(1–42) peptide due to the formulation of the antibody–antigen complex. These data confirm the selectivity of the fabricated sensor to the Aβ(1–42) peptides.

The stability of the immunosensor was also examined by storing the antibody modified SPGEs in a refrigerator at 4 °C for 1 week. The current response of the prepared immunosensor decreased by 22% after eight days, thus representing an acceptable stability.

#### 3.4.3. Spiked plasma analysis

**Fig. 8:**
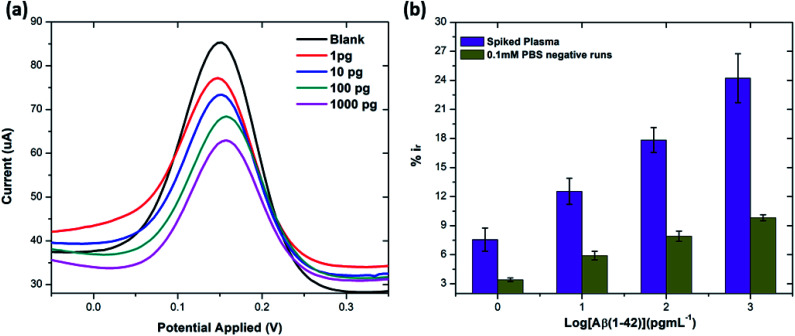
(a) DPV response from spiked concentrations of Aβ(1–42) (at 1, 10, 100 and 1000 pg mL^−1^) in human plasma. (b) Bar chart illustrating the comparison of peak current reduction (%*i*_r_) with exposure to the different concentrations of Aβ(1–42) spiked in human plasma and 0.1 mM PBS (negative runs with respect to each peptide concentration) (*n* = 3).

Blood-based detection of Alzheimer's biomarkers is emerging as a promising alternative to the traditional strategies.^54^ In this study human plasma was first diluted with PBS in the ratio 10 : 100 and later spiked with known concentrations of beta amyloid peptide Aβ(1–42) including 1, 10, 100 and 1000 pg mL^−1^ respectively. The percentage reduction in peak current (%*i*_r_) *vs.* log of peptide concentrations (pg mL^−1^) is shown in Fig. 8. The peak current reduction (%*i*_r_) with exposure to the 0.1 mM PBS was also replotted here to show a comparison with the human plasma results. A significant reduction of DPV signal (up to 27%) in spiked samples was observed as compared to the PBS only samples (up to 9.8%). These results validate the applicability of the proposed method in clinical sample analysis.

## 4. Conclusions

In summary, a simple and rapid label-free electrochemical biosensor, based on a modified graphene screen printed electrode, has been developed for highly sensitive and selective detection of Aβ(1–42). Under the optimized conditions, our proposed immunosensor exhibited excellent analytical performance for Aβ(1–42) with a wide linear range and low detection limit. Antibodies targeted against Aβ(1–42) were immobilised onto the amine surface using EDC/NHS chemistry to obtain preferential antibody orientation attachment. Furthermore, three different concentrations of anti-beta amyloid antibody were used in order to understand the effects that each concentration had on Aβ(1–42) detection sensitivity. The sensor showed negligible response to the non-specific interactions with Tau-352, Aβ(1–40) and APO-E4 proteins. It also showed excellent sensing performance for spiked human plasma samples. Our proposed immunosensor modification method can certainly be used for the detection of other proteins, with optimisation of detection platforms enabling tuning of the sensitivity range. With all these characteristics, the main limitation of our biosensor is its sensitivity towards the water and PBS only samples. Therefore, for future work we are aiming to develop a biosensor with negligible sensitivity towards these fluids.

## Ethical approval

Ethical approval was provided by the local ethics committee for clinical research (Milan, Italy).

## Conflicts of interest

The authors declare no conflict of interest.

## Supplementary Material

NA-003-D0NA00801J-s001
