# Supplementary material for: Graphene based electrochemical immunosensor for the ultra-sensitive label free detection of Alzheimer's beta amyloid peptides Aβ(1–42)
Source: Nanoscale Adv. 2021 Feb 20;3(8):2295–304. doi: 10.1039/d0na00801j (PMC9419744; doi:10.1039/d0na00801j)
Supplement: NA-003-D0NA00801J-s001 [file NA-003-D0NA00801J-s001.pdf]

Electronic Supplementary Material (ESI) for Nanoscale Advances.
This journal is © The Royal Society of Chemistry 2021

Supplementary Information

Graphene based electrochemical immunosensor for the ultra-sensitive label free detection of Alzheimer's beta amyloid peptides Aβ(1-42)

Hina. Y. Abbasi[a,b]*, Zari Tehrani[a,b], Anitha Devadoss[a,b], Muhammad Munem Ali[a] and Owen. J. Guy[a,b]*

[a]College of Engineering, Centre for NanoHealth, Swansea University, Singelton Park, Swansea, SA2 8PP, Wales, United Kingdom

[b]Department of Chemistry, College of Science, Swansea University, Singelton Park, Swansea, SA2 8PP, Wales, United Kingdom

*Corresponding authors, Email: h.y.abbasi@swansea.ac.uk

o.j.guy@swansea.ac.uk

S.1 – Antibody incubation time optimization:

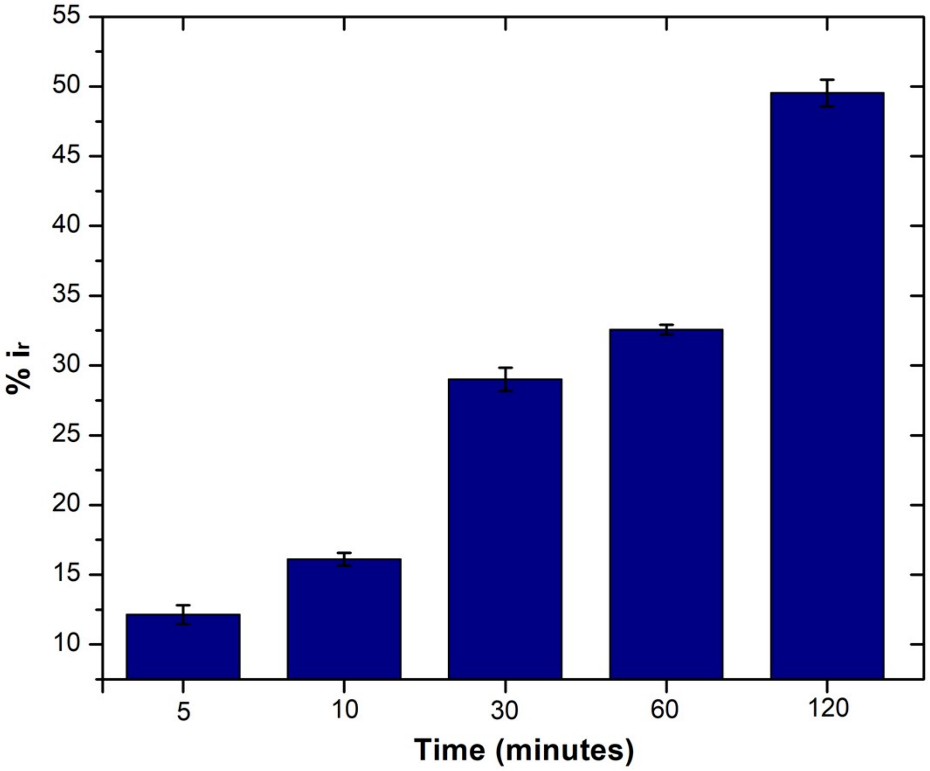

Figure S1: Bar chart representing the antibody incubation time vs the percentage reduction in the peak current obtained from DPV analysis (n=3).

60µg anti-beta amyloid antibody was drop casted over the sensor surface at 4°C for 5, 10, 30, 60 and 120 minutes, respectively. The DPV responses obtained showed the decrease in peak current with increasing the incubation time. Therefore, 30 minutes of drop casting was chosen as the optimum incubation time.

S.2 - Electropolymerization:

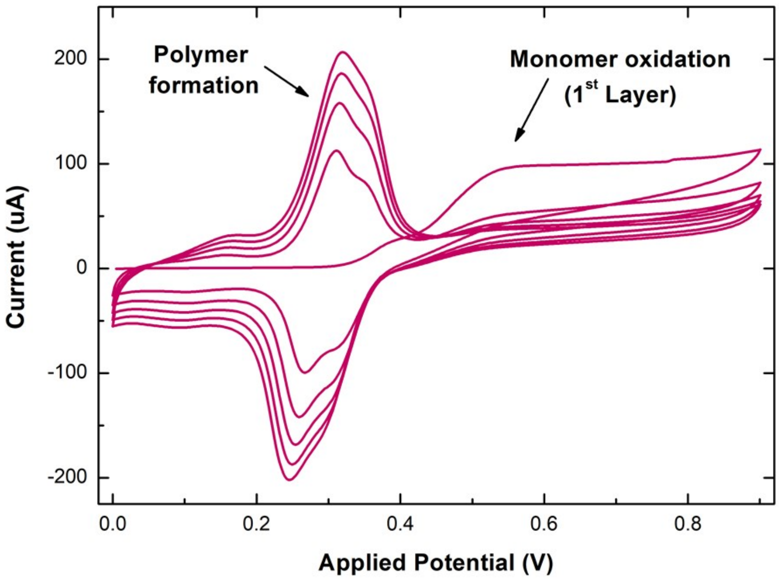

Figure S2: Electropolymerization of pDAN for after 5 repeated voltammetry cycles. Cyclic voltammetry (CV) was carried out between 0V to 0.9V with 50mVs^{-1} scan rate.

S.3 - Electrochemical Analysis:

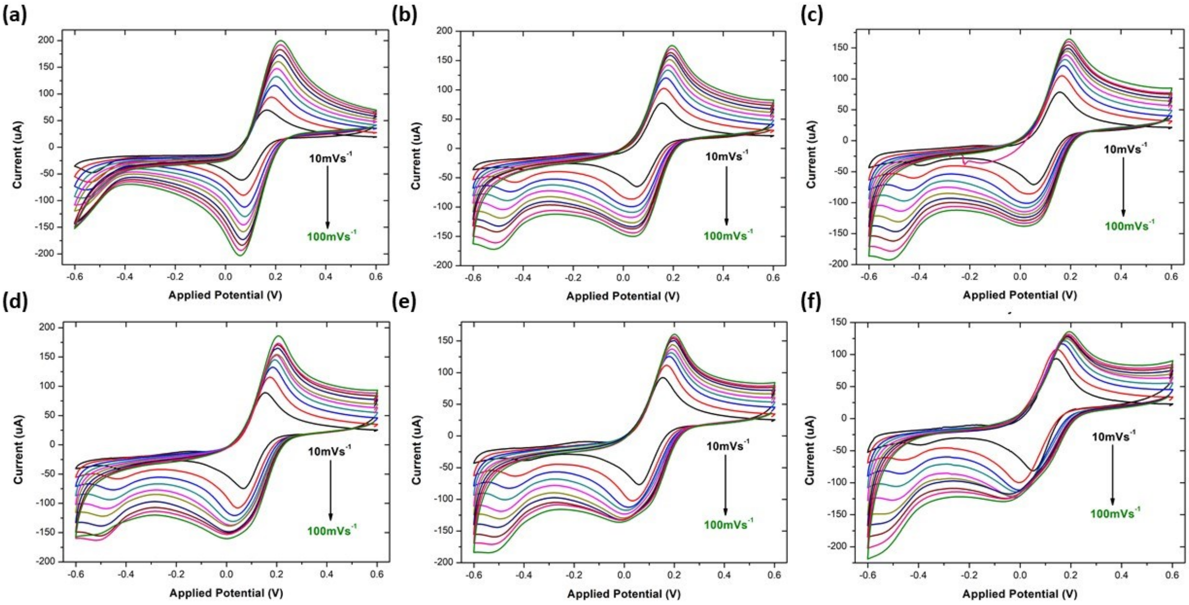

Figure S3: Cyclic Voltammograms of Blank and pDAN modified SPGEs in 5mM $[Fe(CN)_6]^{3-/4-}$ redox couple at scan rates between 10 - 100mVs^{-1} (a) Blank SPGE (b)1 Cycle pDAN SPGE (c)2 Cycles pDAN SPGE (d)3 Cycles pDAN SPGE (e)5 Cycles pDAN SPGE and (f) 10 Cycles pDAN SPGE.

The Blank SPGE (a) shows typical oxidation and reduction peaks at 0.18V and 0.05V, which are attributed to $[Fe(CN)_6]^{3-/4-}$redox reactions. Whereas, polymer DAN modified SPGEs (b-f) exhibited a slight shift in the redox potentials with increasing scan rates which possibly show the occurrence of further chemical reactions [1].

A strong linear dependence of the peak current with the square root of the scan rate was observed, which attests to a diffusion-controlled process on pDAN modified electrodes. The linearity dependence is also confirmed by the regression coefficient (R^2) value (Figure S4). The linearity indicates a typical diffusion-controlled quasi reversible process.

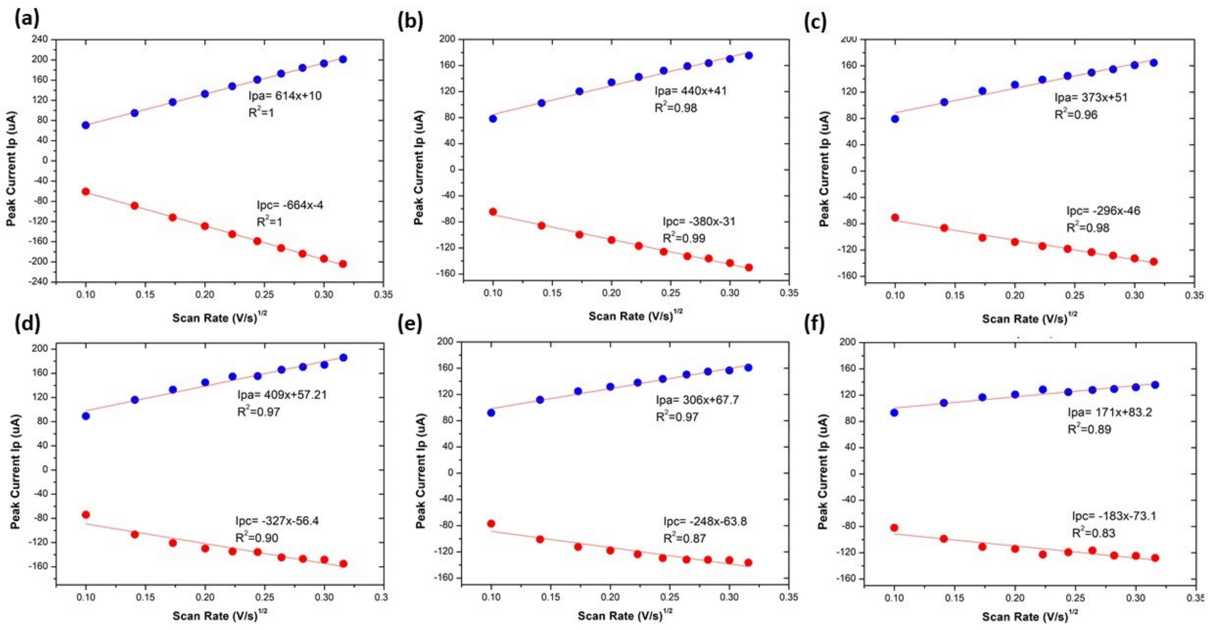

Figure S4: Anodic and Cathodic Peak Current (Ip) values from CV curves of Blank and pDAN modified SPGEs (a) Blank SPGE (b)1 Cycle pDAN SPGE (c)2 Cycles pDAN SPGE (d)3 Cycles pDAN SPGE (e)5 Cycles pDAN SPGE (f) 10 Cycles pDAN SPGE.

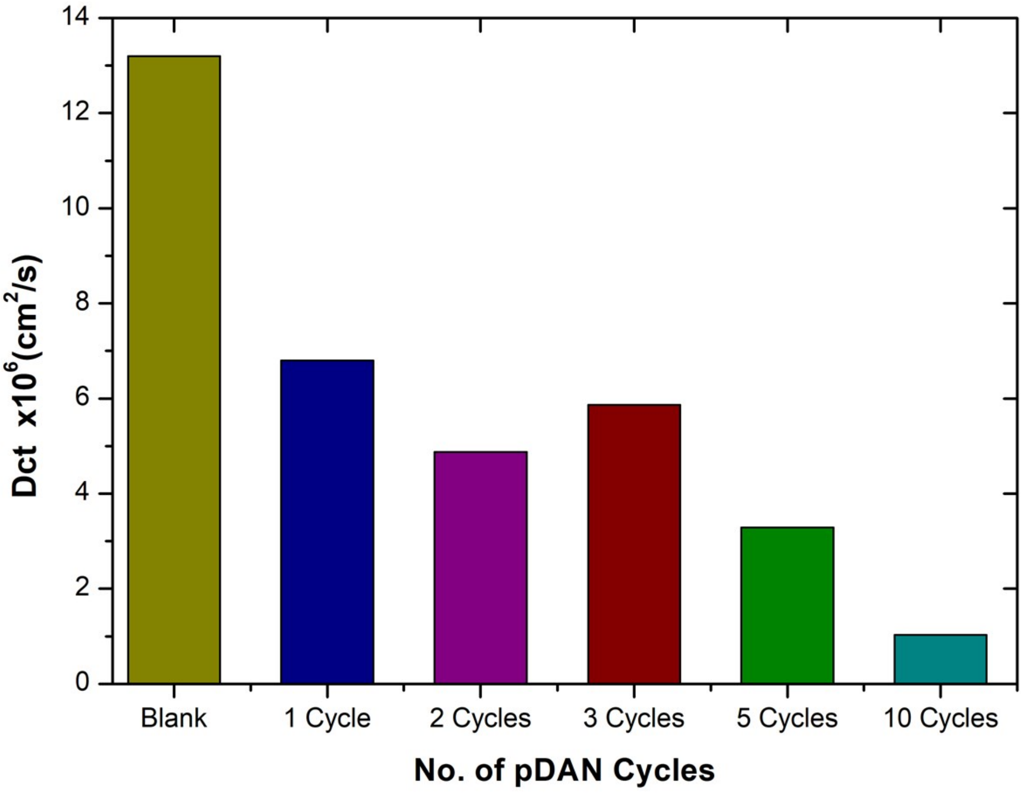

Figure S5: Bar chart illustrating the change in charge transfer diffusion coefficient (D_{CT}) vs. number of pDAN scan cycles.

No. of Cycles	Area Under the Curve (10^{-7})	Surface Coverage (10^{-11}) mol.cm^{-2}
**1**	1.14046	0.941
**2**	1.27827	1.0548
**3**	1.70687	1.408
**5**	1.90891	1.575
**10**	2.388	1.9705

Table S1: Surface coverage values calculated by integrating the reduction part of CV curves obtained after FeCOOH attachment on the SPGEs at different scan cycles.

S.4 - Structural and Chemical Analysis:

Element \ Sample	Atomic Concentration, %	
	**Blank SPGE**	**pDAN Modified SPGE**
**Carbon**	95.11	75.81
**Oxygen**	04.89	14.29
**Nitrogen**	00.00	09.89

Table S2 (A): List of Elemental Concentrations of Blank SPGE and pDAN-Modified SPGEs.

C Component \ Sample	Atomic Concentration, %	
	**Blank SPGE**	**pDAN Modified SPGE**
$C_{aromatic}$	82.08	47.06
$C_{aliphatic}$	03.01	27.66
**C-N**	04.98	14.18
**C-O**	03.66	06.39
**C=O/C=N**	04.31	03.52
**O-C=O/N-C=O**	01.96	01.19

Table S2 (B): List of Carbon Component Concentrations of Blank SPGE and pDAN-Modified SPGEs.

N Component \ Sample	Atomic Concentration, %	
	**Blank SPGE**	**pDAN Modified SPGE**
**C-N**	00.00	91.76
**C-NH**	00.00	04.45
**N-H**	00.00	03.79

Table S2 (C): List of Nitrogen Component Concentrations of Blank SPGE and pDAN-Modified SPGEs.

S.5 - Evaluating the Electrochemical immunosensor performance:

**Aβ antibody Concentrations (µgmL^{-1})**	**Limit of Detection (LOD)**	**Limit of Quantification (LOQ)**
**20**	1.4	4.25
**40**	2.6	7.9
**60**	2.8	8.7

Table S3: List of LOD and LOQ obtained for the electrodes functionalised with different concentration of antibody.

References:

1. Devadoss, A., et al., *Ultrathin Functional Polymer Modified Graphene for Enhanced Enzymatic Electrochemical Sensing.* Biosensors, 2019. **9**(1).
